# Bandwidth Broadening of Piezoelectric Energy Harvesters Using Arrays of a Proposed Piezoelectric Cantilever Structure

**DOI:** 10.3390/mi12080973

**Published:** 2021-08-17

**Authors:** Marwa S. Salem, Shimaa Ahmed, Ahmed Shaker, Mohammad T. Alshammari, Kawther A. Al-Dhlan, Adwan Alanazi, Ahmed Saeed, Mohamed Abouelatta

**Affiliations:** 1 Department of Computer Engineering, Computer Science and Engineering College, University of Ha’il, Ha’il 55211, Saudi Arabia; marwa_asu@yahoo.com; 2 Department of Electrical Communication and Electronics Systems Engineering, Faculty of Engineering, Modern Science and Arts (MSA) University, Cairo 12566, Egypt; 3 Department of Electronics and Communications, Faculty of Engineering, Helwan University, Helwan 11731, Egypt; starshimaa_3011993@yahoo.com; 4 Department of Engineering Physics and Mathematics, Faculty of Engineering, Ain Shams University, Cairo 11566, Egypt; ahmed.shaker@eng.asu.edu.eg; 5 Department of Computer Science and Information, Computer Science and Engineering College, University of Ha’il, Ha’il 55211, Saudi Arabia; md.alshammari@uoh.edu.sa (M.T.A.); k_aldhlan@hotmail.com (K.A.A.-D.); a.alanazi@uoh.edu.sa (A.A.); 6 Electrical Engineering Department, Faculty of Engineering and Technology, Future University in Egypt, New Cairo 11835, Egypt; 7 Department of Electronics and Communications, Faculty of Engineering, Ain Shams University, Cairo 11566, Egypt; m.abouelatta@eng.asu.edu.eg

**Keywords:** bandwidth broadening, energy harvesters, piezoelectric, single-beam cantilever

## Abstract

One of the most important challenges in the design of the piezoelectric energy harvester is its narrow bandwidth. Most of the input vibration sources are exposed to frequency variation during their operation. The piezoelectric energy harvester’s narrow bandwidth makes it difficult for the harvester to track the variations of the input vibration source frequency. Thus, the harvester’s output power and overall performance is expected to decline from the designed value. This current study aims to solve the problem of the piezoelectric energy harvester’s narrow bandwidth. The main objective is to achieve bandwidth broadening which is carried out by segmenting the piezoelectric material of the energy harvester into *n* segments; where *n* could be more than one. Three arrays with two, four, and six beams are shaped with two piezoelectric segments. The effect of changing the length of the piezoelectric material segment on the resonant frequency, output power, and bandwidth, as well as the frequency response is investigated. The proposed piezoelectric energy harvesters were implemented utilizing a finite element method (FEM) simulation in a MATLAB environment. The results show that increasing the number of array beams increases the output power and bandwidth. For the three-beam arrays, at *n* equals 2, 6 mW output power and a 9 Hz bandwidth were obtained. Moreover, the bandwidth of such arrays covered around 5% deviation from its resonant frequency. All structures were designed to operate as a steel wheel safety sensor which could be used in train tracks.

## 1. Introduction

During the past two decades, there have been significant improvements in the development of low-power, small-size, portable and remote devices. Such improvements have caused considerable demand for replacing conventional power sources with unconventional sources. Recent studies have focused on harvesting energy from the ambient. Energy harvesting is essential in applications with an inaccessible environment or where the maintenance cost is high. Some of the scenarios identified in the literature are wireless sensor nodes in remote areas, implanted health trackers, biomedical devices [1,2], and wireless sensor networks on a large scale [3,4].

Energy harvesting is defined as the direct conversion of ambient energy such as vibration, thermal, wind, and solar energy into electrical energy [5,6,7,8]. There are many techniques for harvesting vibration energy [9,10,11,12]. The main method is the piezoelectric technique which gives the highest output power density. Furthermore, it is compatible with most applications. Piezoelectric energy harvesting means converting the input mechanical vibrations into electrical energy using piezoelectric materials. Compared with solar energy harvesters, which may generate hundreds of watts, piezoelectric energy harvesters are used to harvest low energy levels in the range of microwatts to milliwatts. This low energy level is used as a source of energy for low-power electronics.

There are some advantages of piezoelectric energy harvesters over solar energy harvesters, such as ambient vibrations that are primarily available due to the operational conditions of the system. Thus, it does not depend on unstable and unpredictable environmental conditions that can fluctuate with time. Consequently, it is useful in embedded systems, including wireless sensing nodes [13]. It is reported that the worldwide annual benefit of piezoelectric energy harvesters has increased from USD 22 billion in 2012 to USD 37 billion in 2017 [14]. This shows an increasing demand for the applications that use piezoelectric systems.

When a piezoelectric material is subjected to mechanical strain, it generates electric charges where the charge value is proportional to the applied stress [15]. The piezoelectric energy harvester has to be excited at its resonant frequency. At resonance, the maximum power is attained, and a significant vibration response can be produced by a small force [16]. Many techniques have been studied for adjusting the energy harvester’s resonant frequency [17]. The deviation from the resonance condition results in a sudden drop in the generated power [18,19]. Thus, it is crucial to achieve the frequency tuning for the output of piezoelectric energy harvesters.

On the other hand, the main drawback of the piezoelectric energy harvesters is their narrow bandwidth, although a narrow bandwidth is required to give a high output power. Unfortunately, this limited bandwidth makes implementation of the energy harvesting device difficult for it to be suitable for real-life applications [13]. The reason is that most vibrational sources have fluctuating frequencies during their operation. Unfortunately, the maximum output power of the harvester is applied for a small range of frequencies, about 2–3 Hz [15]. The resonant frequency of the harvester also varies from its designed value by 1–5% because of manufacturing processing [16]. Thus, to have a well-functioned piezoelectric energy harvester, its designed bandwidth must cover at least a 5% deviation from its resonant frequency.

Recent studies have focused on solving the problem of the narrow bandwidth for piezoelectric energy harvesters. There are three main techniques that are used to achieve the bandwidth broadening for piezoelectric energy harvesters. The first technique is the resonant frequency tuning [17], which is based on adjusting the stiffness or the preload to the required frequency. The second technique is the multi-modal energy harvesting, which uses a multi-degree of freedom [18]. This technique targets multiple vibration frequencies but at different times. The third technique is based on constructing an array from the piezoelectric cantilever with a different resonant frequency. It is used to harvest electrical energy from different frequencies at the same time [19]. This technique is the most widely used one due to its flexibility of adding or eliminating a single piezoelectric cantilever beam. Each piezoelectric cantilever beam in the array has a different resonant frequency, and each cantilever beam is adjusted by changing the applied proof mass or changing the dimension of the cantilever beam geometry [20]. Unfortunately, in many cases, the array bandwidth does not increase by increasing the number of cantilever beams. The reason is that when the number of array beams increases, the resulting output power becomes less than half the maximum power of the overall system. As the output power is a function of the frequency, any small change in the technological parameters of the structure, such as its length or thickness, causes a significant shift in its resonant frequency [21].

There are other recent research works which handle broadening the piezoelectric energy harvester bandwidth, such as acquiring the bandwidth broadening by tuning the harvester proof mass [22]. Broadband energy harvesting is achieved by integrating the piezoelectric patches with a thermally induced bistable plate. Such a plate is made of the functionally graded carbon nanotube reinforced composite (FG-CNTRC) [23]. Furthermore, a T-shaped piezoelectric energy harvester (TPEH) with an internal resonance and multimodal vibration characteristics is proposed. The main objective of such a proposed piezoelectric harvester is to internally adjust the resonant frequency [24]. Another technique is carried out by introducing nonlinear mechanical dynamics [25]. Bandwidth broadening of the piezoelectric energy harvest can be achieved by immersing the moving mass in a liquid medium [26].

A traditional single-beam piezoelectric cantilever structure as in [27] is a rectangular cantilever. For this traditional single-beam piezoelectric cantilever, the length of both cantilever beams changes to achieve frequency tuning. This conventional structure has major drawbacks concerning accomplishing accurate frequency tuning for piezoelectric energy harvesters. Firstly, it has only two output power peaks through its frequency range with a relatively high difference, 0.02 µW [27]. Meanwhile, to achieve effective frequency tuning, the peaks of each output power through the structure frequency range should ideally be equal. The second drawback of such a structure is that the gap between resonant frequencies at which the peak output power occurs is large, 8 Hz. Practically, it has to be around 1 Hz.

In this paper, a proposed single-beam piezoelectric cantilever structure is investigated. The theory of operation of such a structure is based on segmenting its piezoelectric material into *n* segments. To achieve a fixed output power and bandwidth of the proposed structure, the total length of piezoelectric material, *L_p_*, and the cantilever beam length, *L*, of the structure are set to constant values. To acquire a slight shift in the structure resonant frequency, the length of the piezoelectric material segments is changed. The simulations are carried out for the proposed structure with two segments using FEM simulations in a MATLAB environment. The simulation results show that the output power and bandwidth of the proposed structure are nearly constant at different piezoelectric material segments length. The proposed structure resonant frequency is slightly shifted at each different length of the piezoelectric material segment. Three arrays are constructed from the proposed single beam piezoelectric cantilever structure: two beams, three beams, and six beams array of *n* equals 2. The frequency response of such arrays is also simulated. The output power and bandwidth of such arrays increase when the number of array beams increases. They satisfy a promising bandwidth broadening concerning the operation of the piezoelectric energy harvesters.

The rest of this work is organized as follows; Section 2 introduces a modified version of the traditional structure showing its advantages over the conventional one; Section 3 illustrates the proposed single beam piezoelectric cantilever structure with its theory of operation and performance; Section 4 demonstrates the constructed arrays from the proposed structure as well as the overall results; the conclusion is drawn in Section 5.

## 2. Modified Traditional Single Beam Piezoelectric Cantilever

**Figure 1:**
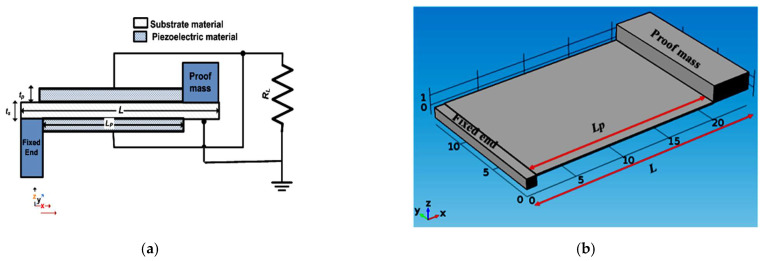
(**a**) Demonstration for the modified traditional structure and (**b**) 3D view of the modified traditional structure.

Figure 1a demonstrates the modified version of the traditional single-beam piezoelectric cantilever structure. It is a rectangular cantilever that has a fixed-proof mass. The length and width of the cantilever beam are denoted as *L* and *W*. The substrate of the cantilever beam is made from steel with a thickness of *t_s_*, as the steel substrate is considered better than silicon in fabricating piezoelectric energy harvesters [28,29]. The piezoelectric material, which is deposited on the cantilever beam, is lead zirconate titanate (PZT_5_H) [28] with a thickness of *t_p_*. In this modified structure, the crystalline piezoelectric material is used as it has the advantage of generating higher output power [28]. To make the best use of the piezoelectric material, it must not be deposited beneath the proof mass and the fixed end. Thus, the piezoelectric material length (*L_p_*) is equal to the total length of the cantilever beam (*L*) minus the summation of both proof mass and the fixed end lengths. The piezoelectric cantilever structure is designed to operate as a steel wheel safety sensor which could be used in train tracks [30].

In the presented design, the proof mass is assumed to be 4 mm in length, and the fixed end is assumed to be 1 mm in length. Therefore, *L_p_* equals (*L* − 5) mm. The piezoelectric material is deposited on the top and the bottom sides of the cantilever beam, a bimorph structure. It is worth mentioning that it generates higher output power compared to the unimorph structure [31,32]. Figure 1b shows a three-dimensional (3D) view of the modified traditional structure.

The technological parameters of the structure shown in Figure 1 were selected as a case study to investigate its performance [33]. The dimensions considered for this case study are summarized in Table 1. In comparison with the traditional structures, the modified structure has only one cantilever beam. As the structure consists of a single beam piezoelectric cantilever, its resonant frequency is inversely proportional to *L*^1.5^. However, the output power of this structure is directly proportional to *L*^1.5^ [34].

There are two advantages of the modified structure in comparison with the traditional one. First, this modified structure uses a single cantilever, so it needs only half the traditional structure volume. Accordingly, it is expected for the modified structure to have a lower fabrication cost than the traditional one. Second, the modified structure uses the crystalline PZT_5_H piezoelectric material, enabling a higher output power generation [28]. Another advantage of the modified structure is that it does not use piezoelectric material beneath its proof mass and the fixed end, which results in no waste in the piezoelectric material, which is responsible for output power generation. Additionally, the modified structure is designed to be bimorph, generating higher output power in analogy to unimorph [31,32].

**Figure 2:**
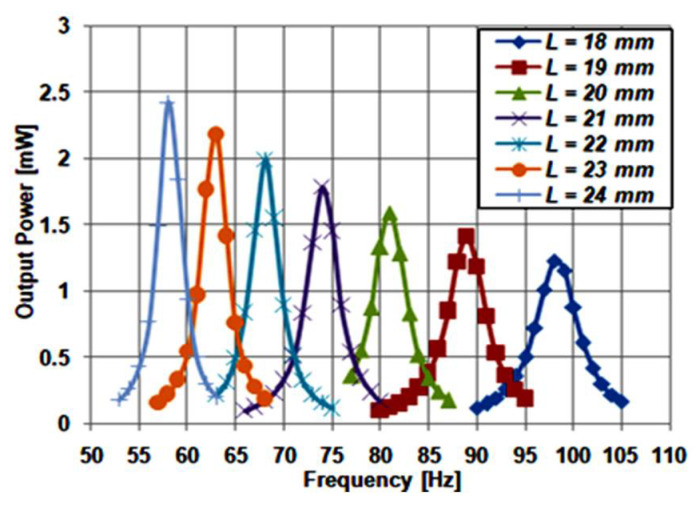
Modified structure frequency response at different lengths.

Next, to investigate the performance of the mentioned design, the influence of varying the cantilever beam length (*L*) on the modified traditional structure performance was simulated. The nominal value of *L*, which is used as a reference, is taken to be 21 mm. All other dimensions of the structure are fixed, as stated in Table 1. To investigate the impact of varying the length on the performance, a simulation was carried out for different values of *L*; ranging from 18 to 24 mm. Figure 2 illustrates the simulation results of the structure frequency response for different values of *L*. It is evident from the Figure that by increasing *L*, the resonant frequency and bandwidth decreased, but the output power increased.

Multiple peak output power at multiple close frequencies must occur through the frequency range to achieve an accurate frequency tuning for the piezoelectric energy harvester. The results of the modified structure were not promising regarding its frequency tuning. However, it still had the advantages of having lower volume and an expected lower fabrication cost compared to the traditional structures. The modified structure gave higher output power as it used the crystalline PZT_5_H instead of the polyvinylidene fluoride (PVDF) piezoelectric material [27]. In the next section, a proposed single-beam piezoelectric cantilever is proposed, where more advantages towards an optimized operation are accomplished.

## 3. Proposed Single-Beam Piezoelectric Cantilever

In this section, a proposed single beam piezoelectric cantilever structure is presented along with its theory of operation and the advantages of such a structure in comparison with the modified and traditional structures. The resonant frequency, output power, bandwidth, and frequency response of the proposed structure are simulated and the simulation results are presented.

### 3.1. The Main Structure and Theory of Operation

The piezoelectric energy harvester is modeled using the mass-spring-damper model. The model is valid for parallel and series connection of the piezoelectric layers. It is used to get the resonant frequency, displacement of end mass and generated voltage across the resistive load. In addition, the effect of the load on both the resonant frequency and the output power is included. In this model, both the base displacement caused by the input vibration and the displacement of the end mass relative to the vibrating base are related by the end mass equation of motion [35]. Such an equation includes the generated voltage, the ratio of the mechanical damping and the natural frequency of oscillation [36,37]. Concerning the mechanical and electrical coupling, in the electrical domain, the equivalent circuit equation with mechanical coupling is derived using piezoelectric constitutive equations. The equation of motion of the end mass and the electrical circuit equation of the piezoelectric energy harvester are called the governing equations of electromechanical coupling. All the required equations to well define such a model are illustrated and explained in detail in Reference [35]. The validation of the model has also been performed by comparing its results vs. the finite element method (FEM) simulator which is used for simulating our proposed structure.

**Figure 3:**
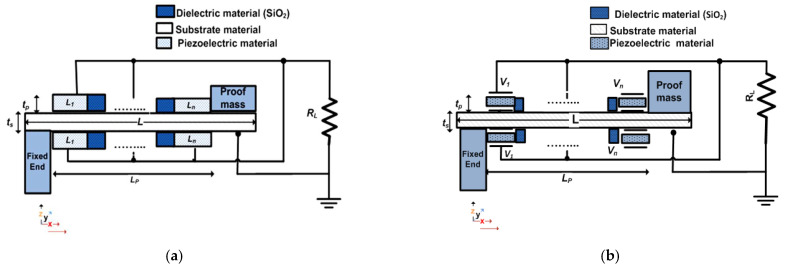
(**a**) Demonstration of the proposed structure with *n* piezoelectric material segments and (**b**) electrical connection.

Figure 3 demonstrates the proposed single-beam piezoelectric cantilever structure along with its electrical connection. The structure has the same technological and physical parameters as the modified structure described in Section 2. Comparing Figure 3a to Figure 1a, the main difference between the proposed and the modified structures is that the piezoelectric material length, *L_p_*, of the structure is split into *n* segments, from *L*_1_ to *L_n_*. Such segments are separated by a dielectric material used to guarantee the mechanical separation and the electrical connection between piezoelectric segments. The used dielectric material is silicon dioxide (SiO_2_). The length of SiO_2_ is selected with a reasonable value not to waste the useful length of the piezoelectric material. In this design, the SiO_2_ length is set to 0.5 mm. The proposed structure has two terminals: the structure substrate and the metal electrode layer deposited above the piezoelectric material [38].

As depicted in Figure 3a, the main objective of the proposed structure is to split its piezoelectric material into *n* segments. From the theory of operation of the rectangle single-beam piezoelectric cantilever structure, the stress has a maximum value at the structure’s fixed end. It gradually decreases until it reaches a minimum value at the free end of the structure [39]. Therefore, the voltage generated by the piezoelectric material has its maximum value at the fixed end *L*_1_ and its minimum value at the free end *L_n_*. To achieve an accurate frequency tuning, the proposed structure’s resonant frequency has to shift slightly. In addition, both its output power and bandwidth have to stay fixed. To satisfy a slight frequency shift, the length of the proposed piezoelectric material segments must change. To have a nearly fixed output power and bandwidth at each subtle frequency shift, the total length of the piezoelectric material, *L_p_*, and the structure cantilever beam length, *L*, must remain constant. This objective is achieved by using the proposed structure.

Further investigations of Figure 3b indicate that all *n* piezoelectric material segments appear as parallel batteries with multiple voltages, from *V*_1_ to *V_n_*. Each segment generates a certain output voltage. Thus, from *L*_1_ to *L_n_*, there are generated voltages from *V*_1_ to *V_n_*; with *V*_1_ having the highest voltage and *V_n_* having the lowest voltage. The piezoelectric material segment *L*_1_ applies a voltage on *L*_2_. As a result, *L*_2_ vibrates by the effect of the voltage applied from *L*_1_. This operation is repeated till it reaches *L_n_*. Therefore, the resonant frequency of the structure changes by the effect of segmenting its piezoelectric material. Both *L_p_* and *L* do not change. Thus, the output power and bandwidth of the structure remain constant. To get the total output power of the structure, as the *n* piezoelectric segments are electrically connected in parallel, the voltages of all the piezoelectric segments are added. Consequently, the total output power is achieved. This qualitative analysis is verified quantitatively in the next subsection.

There is much research concerning designing and optimizing the piezoelectric energy harvester’s load. Such an issue is very important in adjusting, controlling and optimizing the structure’s performance [40,41,42,43]. Regarding our proposed structure, in this phase, our main concern was to evaluate and verify our proposed structure and its performance in achieving the bandwidth broadening. The used load in this work is the resistive load which equals 35 kΩ. Such a resistive load value is designed to match the impedance of the structure to maximize its output power. The required matching impedance is calculated as [44,45]:

(1) $$R_{\mathrm{matched}}=\frac{1}{2\pi f_{\mathrm{res}}C_{p}}$$

where *C_P_* is the capacitance of the piezoelectric layers, and *f*_res_ is the resonant frequency, which is 80 Hz. The capacitance of the piezoelectric material is calculated based on the layer dimensions, including in-plane area and thickness. It is given by:

(2) $$C_{p}=\frac{\varepsilon_{r}\varepsilon_{o}WL}{t_{p}}$$

where *W* is the width (14 mm), *L* is the length (16 mm), *t_p_* is the thickness (0.06 mm), $\varepsilon_{o}$ = 8.854 × 10^−12^ F/m is the permittivity of free space, and $\varepsilon_{r}$ relative dielectric constant of the piezoelectric layers. Therefore, the ideal matched load, *R*_matched_, for the proposed structure is 35 kΩ. The performance of the proposed structure is simulated in the next sections with this matched resistor. Such matched resistive load, as a proof of concept, is sufficient for this phase of our design.

### 3.2. Effect of Changing the Length of Piezoelectric Material Segments

**Figure 4:**
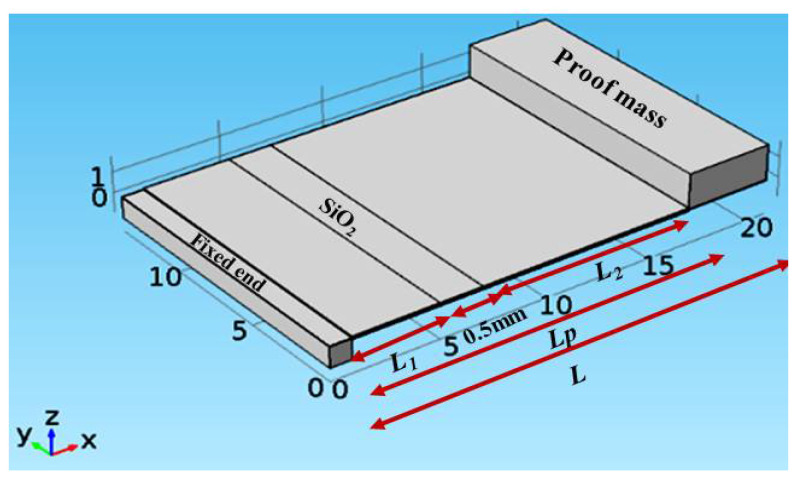
3D representation of the proposed structure with *n* = 2.

In this subsection, the impact of changing the length of the piezoelectric material segments on the proposed structure performance is presented. This study is carried out when the piezoelectric material of the proposed structure is split into two segments, *n* = 2, as shown in Figure 4. To illustrate the effect of changing the length of piezoelectric material segments on the performance, the simulation was carried out for different values of *L*_1_ *L*_2_ = *L_p_* − *L*_1_ − 0.5 mm, where 0.5 mm is the length of SiO_2_. The structure length, *L*, used in this study is 21 mm. All the other dimensions of the structure are the same as the case study presented in Section 2.

**Figure 5:**
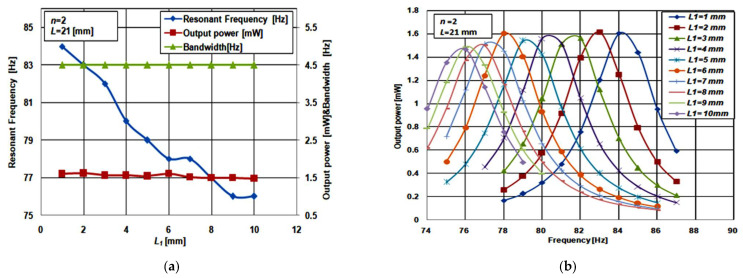
(**a**) Effect of changing *L*_1_ on the resonant frequency, output power and bandwidth and (**b**) frequency response at various values of *L*_1_.

Figure 5a shows the simulation results of the impact of changing *L*_1_ (and thus *L*_2_), on the proposed structure resonant frequency, output power, and bandwidth. By increasing *L*_1_ (and thus decreasing *L*_2_), the structure bandwidth and output power were nearly constant while the resonant frequency was slightly decreased. Additionally, Figure 5b depicts the frequency response at the different values of *L*_1_. These results clarify that the accurate frequency tuning of piezoelectric energy harvester can be achieved using the proposed structure. To emphasize the effect of changing *L*_1_ and *L*_2_ on the structure performance, the same simulations were repeated for the other two values of *L* = 18 and 24 mm at *n* = 2; the same behavior was observed. Thus, the effect of changing *L*_1_ is the same for different values of *L*.

## 4. Arrays of the Proposed Single-Beam Piezoelectric Cantilever Structure (*n* = 2)

**Figure 6:**
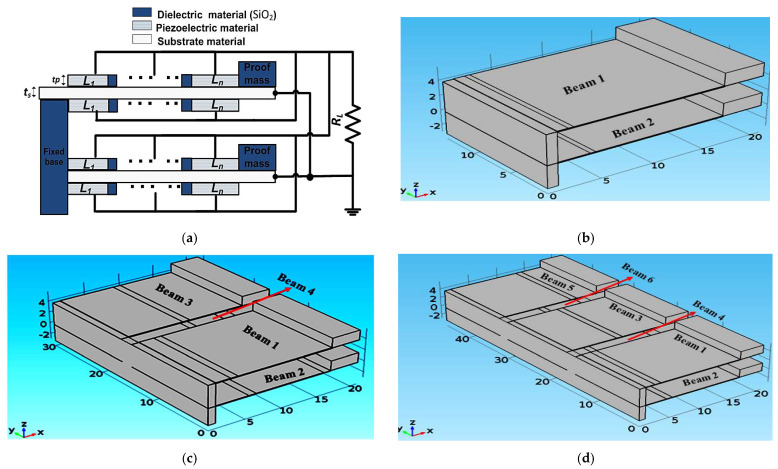
Constructed arrays of the proposed single-beam piezoelectric cantilever structure: (**a**) Demonstration for the two-beam array of the proposed structure at *n* piezoelectric material segments; (**b**) 3D structure of two cantilever beams array; (**c**) 3D structure of four cantilever beam array; and (**d**) 3D structure of the six beams array.

This section presents three arrays of the proposed single beam piezoelectric cantilever structure, as illustrated in Figure 6. The three arrays were constructed from two, four, and six cantilever beams of the proposed structure presented before for *n* = 2. All the arrays were constructed for *L* = 21 mm. Figure 6a shows a demonstration of the array constructed from two cantilever beams of the proposed structure for *n* piezoelectric material segments. The cantilever beams are designed to be connected vertically. This vertical structure requires less area and thus, less fabrication cost is expected than for the horizontal structure. The 3D views of the studied arrays are also shown in Figure 6.

### 4.1. Two Piezoelectric Cantilever Beam Array

Figure 6b shows the 3D structure of the array constructed from two cantilever beams of the proposed structure. The two beams are vertically connected, where the main aim of the design of the two beams array is to achieve a broad and smooth bandwidth. Thus, the array output power has to be smooth and continuous throughout its entire bandwidth. To have a well-functioning piezoelectric energy harvester, the array bandwidth must cover at least a 5% deviation from its resonant frequency [15,16]. By satisfying these requirements, the array achieves a promising bandwidth boarding. These design requirements are met by adjusting *L*_1_ in both beam 1 and beam 2. *L*_1_ is responsible for the frequency shift in the resonant frequency. In the array design, *L*_1_ of beam 1 is fixed at 1 mm to set the end of the frequency range (75 Hz to 88 Hz) at a constant value. To determine the optimum length *L*_1_ for beam 2, which gives the broader array bandwidth at a continuous output power, its value is swept from 2 mm to 7 mm.

**Figure 7:**
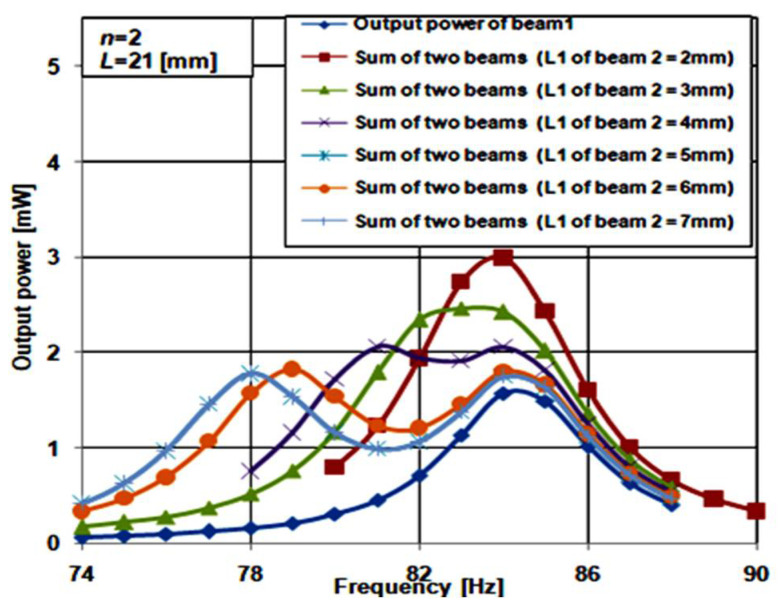
Frequency response of beam 1 and summation of beam 1 and beam 2 (*L*_1_ of beam 2 varies from 2 mm to 7 mm).

Figure 7 shows the frequency response of beam 1 at *L*_1_ = 1 mm. The summation of the frequency response of beam 1 and beam 2 while sweeping *L*_1_ for beam 2 from 2 mm to 7 mm is also shown. At *L*_1_ of 2 mm of beam 2, the maximum output power of the array was 3 mW, and the resonant frequency was 84 Hz. At the same time, the array bandwidth was 5 Hz, in a range from 81.5 Hz to 86.5 Hz. This bandwidth covers a 2.1% deviation from the resonant frequency. The array output power was continuous through its bandwidth. At *L*_1_ of 3 mm of beam 2, the maximum output power was 2.5 mW, the resonant frequency was 83 Hz, and the bandwidth was 6 Hz in a range from 80.5 Hz to 86.5 Hz. It covers 2.5% deviation from the resonant frequency. Again, the array output power was continuous throughout its bandwidth. As *L*_1_ of beam 2 increased from 4 mm to 7 mm, the output power decreased and became discontinuous through the bandwidth. The optimum length for *L*_1_ of beam 2 was 3 mm. In conclusion, the two beam array at *n* = 2 with the optimum *L*_1_ of beam 2 satisfies an acceptable bandwidth broadening.

### 4.2. Four Cantilever Beam Array

**Figure 8:**
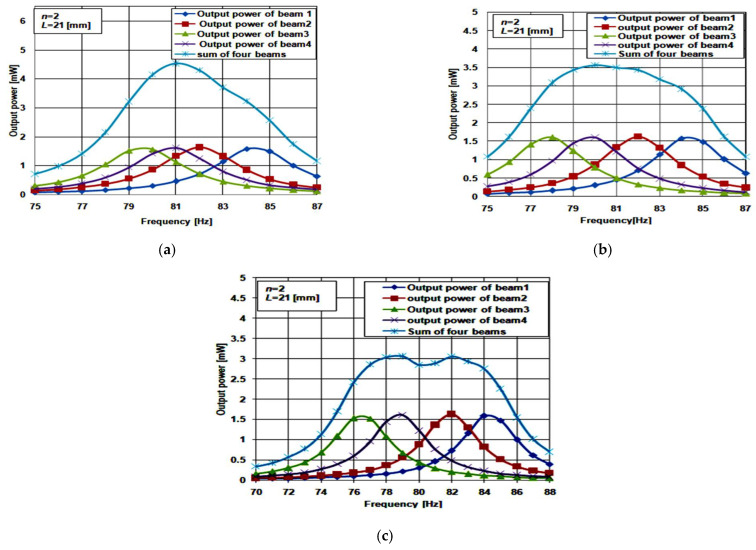
Frequency response of four beams array case 1: (**a**) first case where *L*_1_ for beam 3 and beam 4 equal to 4 and 5 mm; (**b**) second case where *L*_1_ for beam 3 and beam 4 equal to 5 and 7 mm; and (**c**) third case where *L*_1_ for beam 3 and beam 4 equal to 6 and 9 mm.

Figure 6c shows a 3D structure of the array constructed from four cantilever beams of the proposed structure. For beam 1 and beam 2, *L*_1_ has the same values as the two-beam array illustrated before. To determine the optimum value of *L*_1_ for beam 3 and beam 4, three cases were studied. For the first case, *L*_1_ for beam 3 and beam 4 was set to 4 and 5 mm, respectively. In the second case, *L*_1_ for beam 3 and beam 4 were equal to 5 and 7 mm, respectively. For the third case, *L*_1_ for beam 3 and beam 4 were equal to 6 and 9 mm, respectively. The main objective of this study is to achieve an optimum design that achieves the broader bandwidth with continuous output. The bandwidth of the optimum array has to cover at least a 5% deviation in its resonant frequency to have a well-functioning piezoelectric energy harvester. Figure 8a–c show the simulation results for the three inspected case studies.

Regarding the first case (Figure 8a), the array maximum output power was found at 4.5 mW and its resonant frequency was 82 Hz. The bandwidth was 8 Hz, from 78 Hz to 86 Hz. It had a 4% deviation from the array resonant frequency. Thus, case 1 of the four beams array achieved a good bandwidth broadening. Comparing this result with the optimum case of the two-beam array explained before, the bandwidth of the four-beam array increased by 2 Hz, from 6 Hz to 8 Hz. The maximum output power increased by 2 mW, from 2.5 mW to 4.5 mW. Thus, the array output power and bandwidth increased with increasing its beams.

Further, for case 2, as shown in Figure 8b, the array’s maximum output power was 3.5 mW. The resonant frequency was 82 Hz and the bandwidth was 10 Hz, from 76 Hz to 86 Hz. It had a 5% deviation from the resonant frequency. Thus, case 2 achieved a promising bandwidth broadening. Moreover, the array’s maximum output power was 3.1 mW, as extracted from Figure 8c. The bandwidth was 11 Hz and the output power had a 0.2 mW drop at the center of the array frequency response. It was not smooth and continuous throughout the bandwidth. Thus, this case is not recommended for achieving good bandwidth broadening. As a result, case 2 of the four-beam array was the optimum case.

### 4.3. Six Cantilever Beam Array

**Figure 9:**
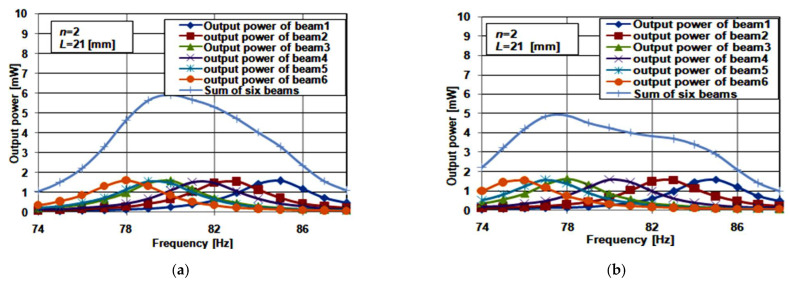
Frequency response six beam array: (**a**) case 1; and (**b**) case 2.

Figure 6d shows a 3D view of the structure of the array constructed from six cantilever beams. For beam 1 and beam2, *L*_1_ had the same values as the two-beam array. Two cases were studied concerning *L*_1_ for beam 3, beam 4, beam 5, and beam 6: for case 1, *L*_1_ for beam 3, beam 4, beam 5, and beam 6 equaled 4, 5, 6 and 7 mm, respectively; while in case 2, *L*_1_ for beam 3, beam 4, beam 5, and beam 6 equaled 5, 7, 9 and 11 mm, respectively. As the length of the piezoelectric material was 16 mm, there was no need to study other cases for the lengths of beam 3, 4, 5, and 6 of the arrays. Figure 9a,b shows the simulation results for the two studied cases of the six cantilever beam array at *n* = 2.

For case 1, the array’s maximum output power was at 6 mW, and its resonant frequency was 80 Hz. The bandwidth was 10.5 Hz, ranging from 75.5 Hz to 86 Hz. It gave a 5% deviation in the array resonant frequency. The array output power was continuous throughout its bandwidth. Thus, case 1 achieved promising bandwidth broadening. Comparing these results with case 1 of the four beam array, the bandwidth increased by 1 Hz, and the output power increased by 1.5 mW. Both the six beam array’s output power and bandwidth increased because of the increasing number of beams.

Finally, Figure 9b shows the maximum output power and bandwidth of case 2 as 4.8 mW and 11 Hz, respectively. The output power was not smooth or continuous through the array’s bandwidth. Thus, this case is not suitable for achieving good bandwidth broadening. As a result, case 1 of the six beam array is the optimum case that achieves a favorable bandwidth broadening.

From Table 2, it is evident that the arrays constructed from the proposed single beam cantilever structure had the advantage that their output power and bandwidth increased when the number of beams increased. The two beam array at *n* equaled 2 satisfied an acceptable bandwidth broadening. The four and six beam arrays at n equal to 2 achieved a promising bandwidth broadening concerning the operation of the piezoelectric energy harvesters. Their bandwidths covered around a 5% deviation in their resonant frequency.

Thus, such promising arrays enable the fabrication of well-functioning piezoelectric energy harvesters. Our structure is better even for two beams, as it has a lower cost. However, the proposed structure in this work requires an optimization of its load. The structure interface circuit, power condition circuit, has to be investigated. Such a circuit plays an effective role in enhancing the piezoelectric energy harvester’s performance. All of such important issues will be taken into consideration in the authors’ future work.

## 5. Conclusions

In this paper, the problem of the narrow bandwidth of the piezoelectric energy harvesters is examined. We suggested a solution for solving the problem by using different array topologies constructed from a proposed single beam piezoelectric cantilever. The proposed idea was numerically evaluated and verified with simulation. First, the proposed single-beam piezoelectric cantilever structure was introduced. The structure was based on segmenting its piezoelectric material to *n* segments, where both the structure cantilever beam and piezoelectric material total length were kept fixed. Simulation results showed that the output from the array constructed from the proposed single-beam piezoelectric cantilever structure provided the necessary bandwidth broadening. Three arrays from the proposed structure were constructed. We found that by increasing the number of array beams, the output power and bandwidth increased. The three-beam arrays produced 6 mW output power and 10.5 Hz bandwidth. The bandwidth of such arrays covered around a 5% deviation in its resonant frequency. These results indicate that promising bandwidth broadening can be achieved when it is used as a piezoelectric energy harvester.

For future work, a genetic algorithm may be proposed to be used as an optimized solution to the three arrays constructed from the proposed single beam piezoelectric cantilever. The structure optimization will be concerned with more investigation of the structure’s technological and physical parameters. In addition, intensive concern will be directed to the design and optimization of the different types of loads for the proposed structure. Such an optimization aims to achieve the optimum output power from the arrays at the broader bandwidth. Such bandwidth has to cover more than a 5% deviation in the array resonant frequency.

Moreover, our structure is a micromachining structure which required a clean room for its fabrication. Thus, to realize our structure practically, the design of the required masks for each photolithography step would be required. It should be mentioned that although our model may overestimate the performance due to some factors that may arise in fabrication, the presented results are promising for the fabrication of such structures to proceed.

## Figures and Tables

**Table 1:** Main design parameters of the modified traditional structure case study.

Parameter	Value (mm)
*L*	21
*W*	14
*t_p_*	0.06
*t_s_*	0.04
*L_p_*	16

**Table 2:** Performance parameters comparison of different arrays of piezoelectric harvesters.

Work	No. Beams	Output Power	Bandwidth (Hz)	Type of Bandwidth
[46]	3	1.1 mW	39.5–44 (4.5 Hz)	Continuous
[47]	4	249 µW	10–20 (10 Hz)	Discontinuous
[48]	8	65.24 µW	10–240 (230 Hz)	Discontinuous
This work	2	2.5 mW	80.5–86.5 (6 Hz)	Continuous
This work	4	3.5 mW	76–86 (10 Hz)	Continuous
This work	6	6 mW	75.5–86 (10.5 Hz)	Continuous

## Data Availability

No new data were created or analyzed in this study. Data sharing is not applicable to this article.
